# Solubility as a limiting factor for expression of hepatitis A virus proteins in insect cell-baculovirus system

**DOI:** 10.1590/0074-02760160153

**Published:** 2016-07-11

**Authors:** Haroldo Cid da Silva, Cristiane Pinheiro Pestana, Ricardo Galler, Marco Alberto Medeiros

**Affiliations:** Fundação Oswaldo Cruz, Instituto de Tecnologia em Imunobiológicos, Laboratório de Tecnologia Recombinante, Rio de Janeiro, RJ, Brasil

**Keywords:** baculovirus, expression, hepatitis A, solubility and vaccine

## Abstract

The use of recombinant proteins may represent an alternative model to inactivated vaccines against hepatitis A virus (HAV). The present study aimed to express the VP1 protein of HAV in baculovirus expression vector system (BEVS). The VP1 was expressed intracellularly with molecular mass of 35 kDa. The VP1 was detected both in the soluble fraction and in the insoluble fraction of the lysate. The extracellular expression of VP1 was also attempted, but the protein remained inside the cell. To verify if hydrophobic characteristics would also be present in the HAV structural polyprotein, the expression of P1-2A protein was evaluated. The P1-2A polyprotein remained insoluble in the cellular extract, even in the early infection stages. These results suggest that HAV structural proteins are prone to form insoluble aggregates. The low solubility represents a drawback for production of large amounts of HAV proteins in BEVS.

Hepatitis A virus (HAV) is a hepatotropic virus that belongs to the genus *Hepatovirus* within family *Picornaviridae*. Its genome consists of approximately 7,500 nucleotides, which encompass a single open-reading frame coding for a single polyprotein. This polyprotein is post-translationally processed into structural and non-structural proteins. The structural proteins of HAV are divided into the polypeptides VP1, VP2, VP3 and VP4, which form the icosahedral capsid of the virus. Non-structural proteins 2B, 2C, 3A, 3B, 3C and 3D are involved in RNA replication and viral polyprotein processing. There appears to be only one serotype, and signiﬁcant antigenic variation has not been detected among different strains (Martin & Lemon 2006).

HAV is the primary etiologic agent of acute viral hepatitis and it is estimated to cause tens of millions of new infections each year worldwide (Wasley et al. 2006). Currently, there are commercially available vaccines against HAV based on inactivated viruses. However, the high cost of production hinders the introduction of these vaccines into the routine of developing countries, where such action could substantially reduce the number of new infections (Pintó et al. 1998).

The neutralisation epitopes of HAV appear to be dependent on the conformation assumed by proteins in the viral particle and, therefore, synthetic peptides and recombinant proteins seem to be less effective in inducing neutralising antibodies (Khudyakov et al. 1999). Nonetheless, it was demonstrated that immunisation with recombinant vaccinia virus carrying coding region for HAV structural polyprotein P1 was able to protect monkeys against challenge with a virulent strain of HAV even in the absence of evidence of cleavage and viral particle formation (Karayiannis et al. 1991). This result creates perspectives to assess whether immunisation with HAV structural proteins could provide protection against infection caused by HAV.

The most studies reporting the expression of HAV structural proteins uses *Escherichia coli* prokaryotic system (Ostermayr et al. 1987, Johnston et al. 1988, Gauss-Müller et al. 1990, Powdrill & Johnston 1991, Baptista et al. 2006). Despite high levels of expression obtained in this system, the HAV proteins were produced predominantly as insoluble aggregates. Furthermore, prophylactic or therapeutic proteins produced in this system require an additional step of purification for removal of lipopolysaccharides (LPS). In this context, the baculovirus expression vector system (BEVS) is an option to express proteins properly folded and LPS-free (Hu 2005).

In spite of advantages of BEVS, the expression of HAV proteins has been little studied in this system (Harmon et al. 1988, Rosen et al. 1993). Given this, further studies are needed to better assess and characterise the expression of HAV proteins in insect cells, targeting the use as vaccine. In the present work, the intracellular and extracellular expression of VP1 protein was evaluated in BEVS. Kinetic expression assay was performed to investigate the dynamic of the distribution of recombinant protein between the soluble and insoluble fractions of lysate. To verify if hydrophobic characteristics would be present in the precursor polyprotein of HAV, the expression of P1-2A protein was also analysed.

The nucleotides corresponding to VP1 and P1-2A genes were chemically synthesised by Integrated DNA Technologies (IDT, USA) based on the sequence of HAV, HM-175 strain (Genbank Accession No. M14707.1). The genes were cloned into the polylinker of pFastBac Dual vector (Thermo Scientific, USA), downstream of the polyhedrin promoter. The expression cassetes were transferred into the baculovirus genome (bacmid) harbored in *E. coli* cell line DH10BAC (Thermo Scientific, USA). The recombinant bacmids were subsequently used to transfect *Spodoptera frugiperda* 9 (Sf9) cells (Thermo Scientific, USA) and thereby generate the baculoviruses containing the VP1 (Bac-VP1) and P1-2A (Bac-P1-2A) genes. The recombinant viruses were titrated by plaque assay. To perform VP1 extracellular expression, the honeybee melittin (HBM) signal sequence was used upstream of the VP1 gene (Bac-HBMVP1). A baculovirus control (bac control) was generated from transfection of Sf9 cells with bacmid without heterologous gene. All procedures to generate recombinant baculoviruses were performed according to the manufacturer’s instructions (Bac-to-Bac Expression System, Thermo Scientific, USA).

Sf9 cells were infected with Bac-VP1, Bac-HBMVP1 and Bac-P1-2A at a multiplicity of infection (MOI) of 5. Seventy-two hours post-infection (p.i.), cells were separated from supernatants and disrupted by SDS reducing buffer [50 mM tris-HCl, 2% (m/v) SDS, 0.1% (m/v) bromophenol blue, 10% (v/v) glycerol, 100 mM 2-mercaptoethanol]. Samples were resolved by 12% SDS-polyacrylamide gel electrophoresis (PAGE) and transferred to a 0.22 mm nitrocellulose membrane. Goat polyclonal to HAV (Abcam, USA) was used as the primary antibody and alkaline phosphatase (AP)-conjugated anti-goat IgG (Abcam, USA) was used as the secondary antibody.

To analyse the kinetic of intracellular distribution, Sf9 cells were infected with Bac-VP1, Bac-HBMVP1 and Bac-P1-2A. These cells were harvested at various time (24-96 h p.i.) and disrupted by lysis buffer (50 mM Tris-HCl pH 7.8, 1% (v/v) Triton X-100 and 150 mM NaCl) for 10 min at room temperature. The lysates were centrifuged at 10,000 g for 10 min to form a pellet of insoluble materials, which were solubilised in buffer containing 50 mM tris-HCl + 2% (m/v) SDS. The protein concentration of the soluble and insoluble fractions was determined by bicinchoninic acid assay (BCA), according to the instructions of the manufacturer (Thermo Scientific, USA). Samples (same amount of protein per lane) were analysed by Western blotting.

The VP1 protein was detected in crude extract of Sf9 infected cells and showed molecular mass of approximately 35 kDa (Fig. 1A). Two smaller bands may indicate protein degradation. As immunoblotting assay was carried out with goat serum rose from infectious particle of HAV, this result indicates that at least some epitopes present in the viral particle were preserved in the recombinant protein.

**Fig. 1:**
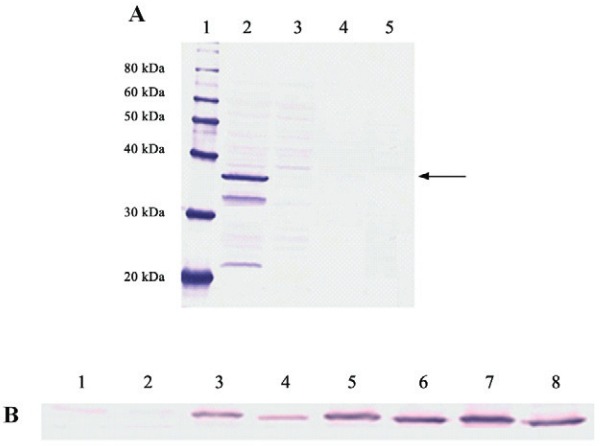
: analysis of expression and kinetics of VP1 intracellular distribution by Western blotting. (A, expression) *Lane 1*, MagicMarkXP Western Protein Standard; *Lane 2*, lysate Bac-VP1; *Lane 3*, lysate Bac control; *Lane 4*, supernatant Bac-VP1; *Lane 5*, supernatant Bac control. (B, kinectics of the intracellular distribution) *Lane 1*, soluble Bac-VP1 24 h p.i.; Lane 2, insoluble Bac-VP1 24 h p.i.; *Lane 3*, soluble Bac-VP1 48 h p.i.; *Lane 4*, insoluble Bac-VP1 48 h p.i.; *Lane 5*, soluble Bac-VP1 72 h p.i.; *Lane 6*, insoluble Bac-VP1 72 h p.i.; *Lane 7*, soluble Bac-VP1 96 h p.i.; *Lane 8*, insoluble Bac-VP1 96 h p.i. The arrow indicates the band that corresponds to the VP1 protein.

To verify the solubility of VP1, it was performed an assay to evaluate the profile of intracellular distribution of this protein. The VP1 was distributed between soluble fraction and insoluble fraction during the periods analysed (Fig. 1B), which suggests the formation of insoluble aggregates.

In order to minimise the formation of insoluble aggregates inside the cells and facilitate the purification procedure, we attempted to direct the expression of VP1 into the extracellular medium. For this purpose, the signal sequence of HBM was inserted upstream of the VP1 gene. It is noteworthy that this signal peptide has been used successfully for expression of several extracellular proteins in BEVS (Tessier et al. 1991, Van der Geld et al. 2002, Kaba et al. 2004). The crude extract and supernatant from cells infected with Bac-HBMVP1 were analysed by Western blotting (Fig. 2A). Two bands were observed in the lysate, between 30 kDa and 40 kDa, which it could correspond to HBMVP1 protein (38 kDa) and to VP1 protein after cleavage of the signal peptide (35 kDa). However, it was not possible to detect the VP1 protein in the supernatant of infected cells as expected.

**Fig. 2:**
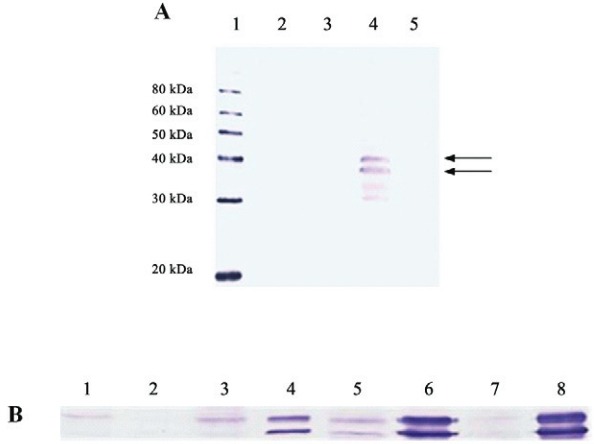
: analysis of expression and kinetics of HBMVP1 intracellular distribution by Western blotting. (A, expression) *Lane 1*, MagicMarkXP Western Protein Standard; *Lane 2*, supernatant Bac-HBMVP1; *Lane 3*, supernatant Bac control; *Lane 4*, lysate Bac-HBMVP1; *Lane 5*, lysate Bac control. (B, kinetics of the intracellular distribution) *Lane 1*, soluble Bac-HBMVP1 24 h p.i.; Lane 2, insoluble Bac-HBMVP1 24 h p.i.; *Lane 3*, soluble Bac-HBMVP1 48 h p.i.; *Lane 4*, insoluble Bac-HBMVP1 48 h p.i.; *Lane 5*, soluble Bac-HBMVP1 72 h p.i.; *Lane 6*, insoluble Bac-HBMVP1 72 h p.i.; *Lane 7*, soluble Bac-HBMVP1 96 h p.i.; *Lane 8*, insoluble Bac-HBMVP1 96 h p.i. The arrows indicate specific bands.

In addition, the kinetics of the intracellular distribution showed that HBMVP1 construction was distributed between the soluble and insoluble fraction of the lysate (Fig. 2B), with high accumulation in the insoluble fraction mainly after 72 h p.i. The low solubility presented by HBMVP1 construction may have contributed to the failure of this approach.

To verify if hydrophobic characteristics would also be present in the precursor polyprotein of HAV, the expression profile of P1-2A protein was evaluated. The P1 region encodes all of the structural proteins of HAV while the 2A nonstructural protein plays an important role in viral morphogenesis being cleaved only at the final stage of maturation of the viral particle (Cohen et al. 2002). The P1-2A protein was detected in the lysate of Sf9 infected cells and showed molecular mass of approximately 110 kDa (Fig. 3A). Lower bands may indicate protein degradation. According to intracellular distribution assay, the P1-2A polyprotein remained insoluble in the lysate for all time analysed (Fig. 3B).

**Fig. 3:**
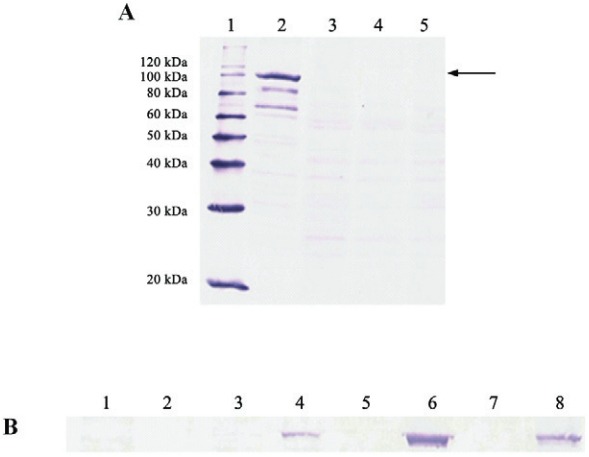
: analysis of expression and kinetics of P1-2A intracellular distribution by Western blotting. (A, expression). *Lane 1*, MagicMarkXP Western Protein Standard; *Lane 2*, lysate Bac-P1-2A; *Lane 3*, lysate Bac control; *Lane 4*, supernatant Bac-P1-2A; *Lane 5*, supernatant Bac control. (B, kinetics of the intracellular distribution) *Lane 1*, soluble Bac-P1-2A 24 h p.i.; Lane 2, insoluble Bac-P1-2A 24 h p.i.; *Lane 3*, soluble Bac-P1-2A 48 h p.i.; *Lane 4*, insoluble Bac-P1-2A 48 h p.i.; *Lane 5*, soluble Bac-P1-2A 72 h p.i.; *Lane 6*, insoluble Bac-P1-2A 72 h p.i.; *Lane 7*, soluble Bac-P1-2A 96 h p.i.; *Lane 8*, insoluble Bac-P1-2A 96 h p.i. The arrow indicates the band that corresponds to the P1-2A protein.

The BEVS has been successfully used to express several viral antigens, including picornavirus proteins (Chung et al. 2006, Cao et al. 2010, Liu et al. 2012). In addition, this technology resulted in two viral vaccines approved for human use, Cervarix^™^ (GlaxoSmithKline) and FluBlok^®^ (Protein Sciences). Cervarix^™^ is a vaccine based on virus-like particles (VLPs) and used to prevent infections caused by human papillomavirus (HPV). FluBlok^®^ is a seasonal influenza vaccine that is comprised of purified recombinant hemagglutinin antigens (Van Oers et al. 2015). The successful expression of viral antigens as well as the history of regulatory approval reinforces the choice of BEVS for this work.

Although BEVS provide a eukaryotic environment for expression of heterologous proteins, the low solubility of HAV proteins could be associated with the need of special requirements for processing (viral protease) and correct folding (human chaperones) of these proteins. However, data obtained from *in silico* analysis using different tools (PROSO, http://mips.helmholtz-muenchen.de/proso/proso.seam; SPpred, http://crdd.osdd.net:8081/sppred/index.jsp) indicated that HAV structural proteins present hydrophobic characteristics. Moreover, we co-expressed P1-2A with virus-encoded 3C protease and observed that VLPs produced also showed low solubility (unpublished observations). So, we believe that the formation of aggregates is related to biochemical characteristics of HAV proteins.

The presence of rare codons, an inefficient internal ribosome entry site (IRES) and the inability to inhibit cellular protein synthesis all contribute to the low replication rate presented by HAV. This feature seems to be important for the virus to overcome the host immune response, since it avoids interferon synthesis and apoptosis (Brack et al. 2002, Pintó et al. 2007). However, the results presented herein allow us to speculate that low replication rate could also be related to the hydrophobic characteristics of the capsid proteins. In this context, the slow replication would be important to avoid aggregation of the viral particles.

Despite the difficulties to obtain recombinant HAV proteins in native form, some approaches can be evaluated to try to improve solubility of these proteins. In this context, the utilisation of cosolvents, detergents or others additives can increase solubility and facilitating proper folding of proteins (Bondos & Bicknell 2003). Another possible approach is to evaluate the effect of fusion tags on solubility of HAV proteins (Esposito & Chatterjee 2006).

In summary, VP1 and P1-2A proteins were expressed in BEVS. The VP1 was detected in both the soluble fraction and in the insoluble fraction of the lysate. The extracellular expression of VP1 was also tried, but it was not successful. The P1-2A was detected only in the insoluble fraction, which may be indicative that HAV structural proteins present low solubility.

The formation of insoluble aggregates may represent an obstacle for obtaining large amounts of HAV proteins in native form. In addition, purification of recombinant proteins from insoluble aggregates usually requires the use of chaotropic agents, which may cause structural alterations during solubilisation and refolding steps (Dill & Shortle 1991). Given this, it is necessary to conduct additional studies to limit the formation of these aggregates and optimise extraction conditions.
